# The warning bell of Dolutegravir resistance: rethinking HIV service models in Africa

**DOI:** 10.3389/fpubh.2025.1667417

**Published:** 2025-09-09

**Authors:** Fausto Ciccacci, Benjamin Welu, Elton Uamusse, Giovanni Guidotti, Yolanda Vaz, Stefano Capparucci, Gianna Iasilli, Dyna Thembo, Paola Scarcella

**Affiliations:** ^1^Department of Biomedicine and Prevention, University of Rome Torvergata, Rome, Italy; ^2^Community of Sant’Egidio, DREAM Program, Meru, Kenya; ^3^Community of Sant’Egidio, DREAM Program, Maputo, Mozambique; ^4^ASL Rome 1, Rome, Italy; ^5^Community of Sant’Egidio, DREAM Program, Rome, Italy; ^6^Community of Sant’Egidio, DREAM Program, Blantyre, Malawi; ^7^LUMSA University, Rome, Italy

**Keywords:** public health, HIV service delivery, resistance testing, Dolutegravir, differentiated service delivery model

## Abstract

The emergence of resistance to Dolutegravir (DTG)—the cornerstone of first-line antiretroviral therapy—marks a critical turning point in the fight against HIV in Africa. Once considered unlikely due to DTG’s high genetic barrier, resistance is now increasingly reported, raising urgent questions about current HIV service models. This Perspective argues that the spread of resistance must be seen not only as a clinical threat but as a warning signal for the broader health system, especially in the wake of post-COVID transformations that have accelerated decentralization, task-shifting, and self-managed care. While such innovations offer benefits, they also risk eroding human connection and clinical oversight, which are essential in managing a complex condition like HIV. Drawing on experience from the DREAM program and other field evidence, we identify four strategic priorities for a renewed response: scaling up differentiated service delivery (DSD), expanding access to resistance testing, strengthening community-based support, and ensuring equitable access to advanced treatment options. These pillars aim to safeguard past achievements while adapting to emerging challenges. We call for urgent, equity-driven action to prevent further spread of resistance and to build resilient, person-centered systems capable of delivering sustainable HIV care in Africa and beyond.

## 1 Introduction

Over the past two decades, Africa has made remarkable progress in the fight against HIV. Millions of people now have access to life-saving antiretroviral therapy (ART), and community-based health systems have played a critical role in expanding care (1). However, a growing concern is casting a shadow over these achievements: the emergence of resistance to Dolutegravir (DTG), the cornerstone of current first-line HIV treatment regimens (2). This phenomenon, once considered unlikely due to DTG’s high genetic barrier, is increasingly reported across different African settings.

This development emerges in a broader context of global health transformation. The COVID-19 pandemic had a direct impact on African health systems through illness and mortality, but perhaps even more significantly, through a series of indirect consequences (3). These included the diversion of resources, disruption of essential services, and a documented increase in mortality from conditions such as tuberculosis (4). In response to the crisis, many health systems also adopted structural changes aimed at reducing crowding in health facilities, minimizing in-person contact, and expanding decentralized or digital modes of care. While such shifts were initially driven by emergency needs, they are now increasingly shaping the permanent architecture of healthcare delivery—including for chronic conditions like HIV.

We are at a pivotal moment. Much has been achieved in terms of access to treatment, community-based care, and health systems strengthening. Yet, critical components of a resilient HIV response are still missing. Over the years, numerous programs have demonstrated that high-quality HIV care is achievable even in low-resource settings (5–7). These experiences underscore that progress is not only possible, but also replicable and scalable when investment, vision, and commitment are sustained. The challenge now is to ensure that these successes are protected, expanded, and adapted to emerging threats.

We observe the spread of DTG resistance in many countries. We described it in Mozambique (8), and similar data have been reported from other countries (2, 9–11). It must be seen not only as a clinical challenge but also as a warning bell for our public health systems. It signals potential weaknesses in how services are currently being designed, delivered, and adapted—often in the name of efficiency or scale. In many countries, we are witnessing a rapid shift toward models that prioritize convenience and cost-containment but risk neglecting the complexity of HIV as both a clinical and social condition.

This paper aims to critically reflect on these emerging service models and argue that excessive simplification—particularly when it leads to the patient’s isolation—may undermine long-term treatment success. Drawing from our experience with the DREAM program and other field-based observations, we highlight key risks associated with “one-size-fits-all” approaches and propose an alternative vision grounded in differentiated care, universal access to resistance testing, and strengthened community engagement.

## 2 Simplification at what cost? Emerging models and their risks

In recent years, efforts to streamline HIV service delivery in Africa have gained momentum, with the goal of increasing coverage, reducing burden on healthcare systems, and enhancing cost-effectiveness. These strategies are particularly relevant in resource-limited settings, where expanding access and optimizing efficiency are critical objectives. Approaches such as decentralization, task shifting, and the use of digital tools are being increasingly adopted to meet these demands. While these innovations offer important opportunities, it is also necessary to consider their implications for continuity of care, patient engagement, and long-term treatment success.

HIV self-testing represents one such innovation. It has the potential to increase testing uptake, particularly among individuals who may be reluctant to visit health facilities or who live in remote areas (12, 13). However, when implemented without sufficient support structures—such as counselling or clear referral pathways—self-testing may lead to challenges. Individuals receiving a positive result in isolation may experience distress or uncertainty about next steps.

Decentralized drug dispensing, through community pharmacies or outreach delivery points, can similarly improve accessibility and reduce congestion at healthcare facilities (14). However, reduced contact with trained healthcare providers may limit opportunities to monitor adherence, detect early signs of adverse effects, or address patient concerns (15). In some cases, patients may receive medications from providers who are unfamiliar with their medical history or who have limited capacity to assess psychosocial factors.

Other evolving practices, such as spacing clinical follow-up visits for stable patients or increasing reliance on task-shifting, may also influence patient experiences (16). Extending intervals between visits can reduce burden on health systems and patients, but may limit the ability to detect changes in health status or emerging support needs (17, 18). Likewise, fragmentation of care and reduced continuity in the provider-patient relationship may affect communication, trust, and the timely identification of problems.

Together, these developments illustrate a broader shift in HIV service models—one that offers clear potential benefits but also introduces new dynamics that warrant careful consideration. Notably, many of these approaches may inadvertently weaken the connection between patients and the health system. As regular contact with providers declines and services become more transactional, care risks becoming depersonalized. This is particularly important in the context of HIV, where stigma, emotional distress, and mental health challenges are prevalent in many contexts (19, 20). Reduced interaction with professionals or peer networks may further intensify these vulnerabilities. Ensuring that simplified models preserve the relational and psychosocial components of care is essential, as their erosion could ultimately compromise outcomes and undermine the efficiencies they aim to deliver.

## 3 Recognizing diversity in HIV care needs

While simplification strategies have played a valuable role in expanding access to HIV services, it is increasingly important to acknowledge the limitations of uniform approaches. HIV is not a uniform disease; it intersects with a broad range of social, economic, and clinical factors that vary across individuals and communities. Standardized models risk overlooking these contextual differences, potentially reducing the effectiveness and equity of interventions.

For example, adolescents living with HIV may encounter challenges such as fear of disclosure, persistent stigma, and difficulties during the transition to adult care—issues that differ significantly from those experienced by older adults or pregnant women (6, 21). Similarly, people living with HIV who have coexisting conditions, such as tuberculosis, diabetes, or mental health disorders, often require more comprehensive and integrated follow-up. Service delivery models that define stability based solely on virologic suppression may fail to identify these important clinical and psychosocial needs.

In addition, many African countries face a rising burden of noncommunicable diseases (NCDs), which now increasingly coexist with HIV. This evolving epidemiological landscape highlights the need for integrated and longitudinal care models that address multiple health issues simultaneously (22–24). Fragmented or disease-specific service structures may struggle to respond effectively to the complex and changing health profiles of people living with HIV.

Responding to the complexity of the HIV epidemic requires a renewed commitment to differentiated service delivery—models that are responsive to the diversity of patient experiences and needs. Incorporating flexibility, cultural competence, and person-centeredness into service design will be essential for sustaining the progress made in HIV care and for closing the persistent gaps that remain in achieving equitable health outcomes.

## 4 Four priorities for a renewed HIV response

**Figure 1:**
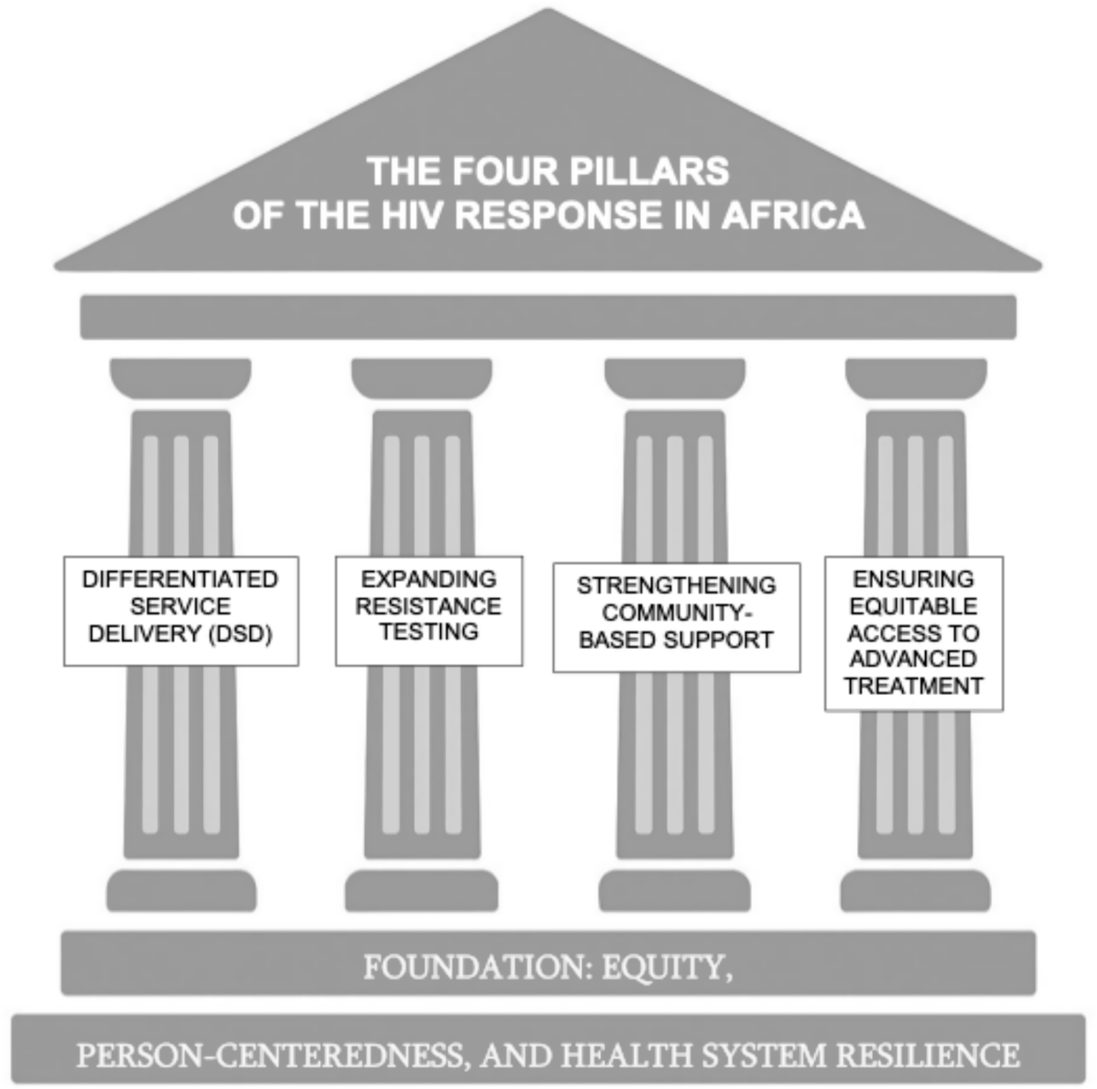
The four pillars of a resilient HIV response in Africa. This diagram illustrates the four key pillars essential to strengthening the HIV response across Africa: (1) Differentiated Service Delivery; (2) Expanding Resistance Testing; (3) Strengthening Community-Based Support; and (4) Ensuring Equitable Access to Advanced Treatments. The entire structure is grounded in a foundation of equity, person-centeredness, and health system resilience—core principles for a sustainable and inclusive response.

Building on the previous sections, we identify four strategic priorities that health systems should focus on to address the evolving challenges of HIV care in Africa. These include the broader implementation of differentiated service delivery (DSD), expansion of access to resistance testing, reinforcement of community-based support systems, and ensuring equitable access to advanced treatment options (Figure 1). Together, these areas offer opportunities to strengthen the resilience and responsiveness of HIV services, particularly in the context of rising drug resistance and shifting epidemiological patterns.

### 4.1 Scaling up differentiated service delivery (DSD)

DSD is an evidence-based approach that adapts HIV services to the diverse needs of individuals and populations (25). It allows for greater flexibility in frequency, location, and provider type while maintaining a person-centered approach. Expanding DSD can help balance efficiency with responsiveness, ensuring that stable patients receive streamlined care while those with complex needs continue to receive more intensive support (26, 27). However, in many African contexts, DSD is currently implemented primarily through the extension of appointment intervals for clinically stable patients—an important but limited step. This approach should be taken further to fully realize the potential of DSD to personalize care. In high-income settings such as Europe, DSD includes differentiated treatment regimens based on individual resistance profiles, offering a model of tailored care that could also benefit African health systems. Implementation must remain context-specific yet more ambitious in scope, and be accompanied by robust monitoring to avoid oversimplification and possible risks (28–30).

### 4.2 Expanding resistance testing

As resistance to Dolutegravir and other antiretrovirals begins to surface, timely detection becomes essential for both clinical management and public health surveillance. Yet, access to resistance testing remains extremely limited in many low-resource settings (31). Investing in affordable, scalable technologies and establishing sentinel surveillance systems can help identify resistance patterns, inform treatment guidelines, and ultimately prevent the wider transmission of resistant strains. Although these tests are currently expensive, previous experiences with viral load monitoring and the scale-up of antiretroviral therapy demonstrate that large-scale implementation and global policy prioritization can significantly reduce costs over time (32, 33). Resistance testing should be seen not only as a technical tool, but also as a strategic investment in the sustainability, effectiveness, and equity of HIV care.

### 4.3 Strengthening community-based support

Community networks—including peer educators, health volunteers, and patient support groups—play a vital role in fostering adherence, reducing stigma, and linking individuals to care (34, 35). As health systems increasingly adopt decentralized and task-shifted models, maintaining human connection and trust becomes even more critical. Strengthening and formalizing these community structures—through training, supervision, and integration with formal health systems—can ensure that simplified service models remain person-centered and supportive. This priority is especially important to address the risk of patient isolation discussed earlier (36, 37). Enhancing community-based support can counteract the depersonalizing effects of simplification, offering meaningful human interaction in contexts where stigma, psychological distress, and mental health challenges already place a significant burden on people living with HIV.

### 4.4 Ensuring equitable access to advanced HIV treatment options

In many African countries, the range of available antiretroviral drugs remains limited compared to high-income settings, where multiple lines of therapy—including new drug classes and long-acting formulations—are widely accessible (38). This disparity constrains clinicians’ ability to respond effectively to treatment failure and emerging resistance. Expanding the portfolio of HIV treatment options in Africa is essential for sustaining long-term viral suppression and improving outcomes, especially in patients with complex resistance patterns or comorbidities.

Achieving this goal requires greater investment, streamlined regulatory processes, and a stronger commitment to global equity in drug access and availability.

Taken together, these four priorities offer a roadmap for reinforcing HIV responses that are both technically sound and socially grounded. They reflect a vision of care that moves beyond minimalistic delivery models, toward systems that are adaptable to context, capable of supporting clinical complexity, and grounded in human connection—systems that are inclusive, resilient, and equipped to meet the diverse needs of people living with HIV.

## 5 Discussion

The emergence of Dolutegravir resistance in African settings highlights a pivotal and urgent moment in the trajectory of the HIV response. While the last two decades have seen substantial progress—particularly in scaling up treatment access, decentralizing care, and strengthening community-based systems—these gains are now being tested by new and complex challenges. Chief among these is the growing threat of resistance to first-line antiretroviral regimens, a development that calls for both technical innovation and a renewed, intentional focus on equity and system resilience.

This paper has identified four strategic priorities that we believe are critical to securing a sustainable and inclusive response: DSD, expanded access to resistance testing, reinforcement of community-based support networks, and the equitable availability of advanced HIV treatment options. These pillars reflect a model of care that is both evidence-based and grounded in the lived realities of people living with HIV in Africa. Ensuring equitable access to high-quality diagnostics and novel therapies must become a standard, not an aspiration. The time lag between the introduction of innovations in high-income countries and their availability in African contexts is no longer acceptable.

Furthermore, the threat of drug resistance underscores the interconnectedness of global health. Infectious diseases do not respect geopolitical boundaries, and neither does resistance. If it is allowed to spread in regions with limited surveillance and therapeutic options, it will eventually reach other parts of the world, potentially undoing decades of progress. The COVID-19 pandemic illustrated in stark terms how fragile and interdependent our public health systems truly are. It also demonstrated that rapid mobilization and coordinated responses are possible when political will aligns with scientific evidence.

Therefore, as we move forward, it is important to support research on the outcomes of simplified HIV service models, especially for vulnerable groups. Studying cost-effectiveness and piloting scalable approaches to resistance testing and differentiated care can guide future implementation. Additionally, ongoing advocacy is needed to ease regulatory barriers and ensure the resources required for broader adoption.

Emerging resistance is more than a clinical concern; it tests the foundation of HIV service delivery. It urges a shift toward adaptable, inclusive systems that leave no one behind. Equity in access and quality is not optional—it is essential to a resilient global response.
